# Data Feature Analysis of Non-Scanning Multi Target Millimeter-Wave Radar in Traffic Flow Detection Applications

**DOI:** 10.3390/s18092756

**Published:** 2018-08-21

**Authors:** Haiqing Liu, Na Li, Deyong Guan, Laxmisha Rai

**Affiliations:** 1 College of Transportation, Shandong University of Science and Technology, Qingdao 266000, China; guandeyong@sdust.edu.cn; 2 College of Electronics, Communication and Physics, Shandong University of Science and Technology, Qingdao 266000, China; sdkjd_lina@163.com (N.L.); laxmisha@ieee.org (L.R.)

**Keywords:** FMCW millimeter-wave radar, traffic flow detection, data feature analysis, grey correlation analysis

## Abstract

The millimeter-wave radar has been widely used in traffic applications. However, little research has been done to install the millimeter-wave radar on the top of a road for detecting road traffic flow at a downward looking direction. In this paper, the vehicle parameters, including the distance, angle and radar cross-section energy, are collected by practical experiments in the aforementioned application scenario. The data features are analyzed from the dimensions of single parameter sampling characteristics and multi-parameter relationships. Further, the correlations of different parameter series are given using the grey correlation analysis method. For millimeter-wave radar used in the traffic flow detection, our work can definitely provide significant support for further intelligent transportation applications, such as vehicle trajectory tracking, traffic flow estimation and traffic event identification.

## 1. Introduction

Real-time road traffic parameter collection and accurate road congestion evaluation are the prerequisites for applying better traffic congestion avoidance strategies to improve the traffic flow [1,2]. The traditional traffic parameter detection methods include: (1) the magneto-electric induction detecting method, in which the section flow data is acquired by loop detectors or geomagnetic detectors placed underground at a certain road section [3,4,5]; (2) the floating vehicle method, which extracts traffic parameters from the trajectory data by intelligent onboard devices, such as bus floating data and taxi floating data [6,7]; (3) the video image detecting methods, for example, the traffic parameter collection by electronic-police camera or using the unmanned aerial vehicles (UAV) [8,9]; and (4) the radar detecting methods.

Among the methods mentioned above, the radar based method has certain advantages. For example, it can avoid ruining roads when deploying the devices and it is affected little by environment, therefore it has been widely used in the field. In the radar detector, the target vehicle is identified by the differences between the transmitting signal and the echo signal and the vehicle speed is calculated based on the Doppler effect. As a maturely applied traffic flow sensor, the radar has been used in urban traffic and expressway management, such as the traffic volume collection and over speed detection. In [10], the radar is installed on the pole at a side of the road and the direction of the antenna beam is perpendicular to the lane direction. With this installation method, the radar can detect the driving status when vehicles moving across the section of the beam. However, it is not able to track the trajectory of the vehicle. In [11], authors use the radar for intersection surveillance in infrastructure cooperative systems. Pedestrians and bikes are accurately detected by using coded pulse modulation and adaptive array antenna. However, they only analyze the echo signal strength for judging pedestrians or bikes and deeper studies on the traffic flow characteristics is missing. In [12], using the non-contact microwave radars, the Fast Fourier Transforms (FFT) method is applied to distinguish from cluster and noise and detect traffic flow characteristics. This work improves the reliability of the traffic detection of each lane better. Further, in [13], authors selected appropriate thresholds for removing noises, reducing FFT side lobes and develop information-aided smart schemes for improving traffic detection of traffic microwave radar detectors in multiple-lane environment for better detection accuracy. In [12,13], the radar is installed on the roadside and detects vehicle targets from orthogonal view to the traffic flows. Similarly, related work has also been presented in [14,15].

Currently, the commonly used radars mainly work at K frequency and X frequency bands [16,17]. These kinds of radars generally use centimeter, decimeter or meter waves. However, considering the natural defect of the Doppler effect, these radars are always insensitive to the low speed objects and they are unable to detect stationary vehicles. Numerous practical examples have shown that, the traditional radars can hardly provide useful traffic data information when the vehicles are traveling under the speed of 20 km/h. The millimeter-wave radar generally works at the 30~300 GHz frequency band and the wave length is 1~10 mm. Since the wave length is between centimeter wave and the light wave, it has the advantages that both the microwave and photoelectric characteristics are integrated. The millimeter-wave radars are initially applied in the military for low-angle tracking, precision guidance, surveying and imaging applications [18,19]. Benefited from the development of military technologies for civil use, the millimeter-wave radar has been widely used in several civilian applications in recent years. Especially, the Frequency Modulated Continuous Wave (FMCW) millimeter-wave radar has been used in road traffic environment as it has the following advantages [20]: (1) The Doppler bandwidth is wide and the Doppler effect is evident, hence the velocity measurement precision is high. (2) The details of objects can be captured sensitively because of the scattering properties of the millimeter-wave radar beam, which has high multi-objective resolution capacity and imaging quality. (3) There is almost no blind zone for the radar range. (4) The radar is with high sensitivity for receiving signal and it has strong anti-interference capability.

As the advantages mentioned above, the echo signal of the FMCW millimeter-wave radar contains two kinds of objects, the moving object with a certain Doppler speed and the stationary object with strong radar cross-section (RCS) energy. The stationary objects are not only the vehicles but also all kinds of surrounding road information, such as road, guardrails, lamp posts and other information, which are collectively known as the background noise. Since the target materials and the reflecting surfaces of the objects are different, the received RCS energy values are also distinct. This feature can be used for distinguishing vehicles and background interferences by setting certain RCS energy thresholds. Currently, the millimeter-wave radar can detect a wide range of intersection and it is also capable for tracking low speed vehicles (0–25 km/h), overcoming the aforementioned shortcomings of the traditional microwave radar. Nowadays, the millimeter-wave radar is mainly used for driving environment perceiving in the advanced driver assistance systems (ADAS) and UAV. The main work related to such radars can be classified into two directions. (1) progress towards hardware and software based on driving environments, such as the antenna optimization and improvement of the target resolution capacity as a lower layer and (2) data mining research for forward obstacle detection subjected to actual applications such as the target identification and trajectory tracking as higher layer.

In [21], authors present a Polarimetry capable antenna to perform radar cross section. The guide posts have a unique RCS signature and it can be used to discriminate them from metal posts measurements of traffic signs at 77 GHz. In order to improve the performance of target detecting in the near zone of antennas, [22] presents a full-wave numerical simulation over popular vehicle types including sedans and trucks. The research identifies various beam widths conditions of phased array antennas to estimate the electromagnetic (EM) backscattering fields. After analyzing the principles of the millimeter-wave traffic radar sensor and the properties of the echo power, a novel background suppression method is proposed in [23]. On the basis of order statistics and coherent averaging, the proposed method noticeably improves the background suppression capacity. In [24], authors proposed a π/2 shift pulse modulation method with a complementary code pair cycle to suppress sidelobe increase. Moreover, in order to improve the angular resolution, a high-resolution direction of arrival estimation involving Tx beam scanning is also presented.

For target identification and trajectory tracking research, [25] proposes a blind spot detection and warning system (BSDWS) using the millimeter-wave radar. The line frequency modulated continuous wave (LFMCW) millimeter-wave radar system is used to monitor the moving targets which are in the blind spot warning area behind a certain vehicle. The work proposes to maintain higher detection and low false detection rate. In [26], authors present their research on information processing of the millimeter-wave radar obstacle in the self-driving car environment sensing module. In the tracking aspect, the method has been added to provide edge information by surrounding obstacles to determine the direction of travel, improving the accuracy and stability of the algorithm. In [27], a fast square root cubature Kalman Filter (CKF) method for automotive millimeter-wave radar target tracking is proposed. In [28], to solve the problems such as false alarm and missing detection in the FMCW radar, an improved multi targets detection algorithm is proposed combining the phase difference and power difference for frequency matching. In ADAS or UAV applications, to overcome the defects of single sensor and improve the target recognition rate, the millimeter-wave radar is also used for data fusion with other sensors, such as the camera [29,30,31,32,33,34,35]. Recently, many scholars have devoted their research on this and definitely it has the potential to become the most important technology in future, with the rapid development of the ADAS and UAV.

As elaborated above, current research on radar for traffic applications can be generalized as follows: On one hand, in the traffic flow detection applications, the applied radars are mainly the 24 GHz microwave radar and they are generally installed on the roadside, detecting vehicle targets from the view orthogonally to the movements of vehicles. On the other hand, the millimeter-wave radar is mainly used for the road environment perceiving in ADAS and UAV applications. The radar is installed onboard for the forward obstacle detection. Little research has been carried regarding installation of the millimeter-wave radar on top of the road for detecting road traffic at a downward direction towards traffic flow. In this paper, we make an attempt that the FMCW millimeter-wave radar is used to detect traffic flow characteristics and analyze the data features. Differing from the onboard installation method for ADAS applications and the orthogonally to traffic flow installation method of traditional radars, we install the radar device above the road, for example, at the cross arm of electronic police devices. In this case, the radar detects the vehicles at a downward looking direction. By practical experiments, we collect the vehicle parameters and analyze the data features, including the sampling characteristics of the distance, angle, RCS energy and the multi-parameter relationships. Further, the correlations of different parameters are given using the grey correlation analysis method. For millimeter-wave radar used in the traffic flow detection field, our work can therefore provide useful support for further intelligent transportation applications, such as vehicle trajectory tracking, traffic flow estimation and traffic event identification.

The rest of the paper is organized as follows. Section 2 introduces the millimeter-wave radar and the experimental setting. In Section 3, the single parameter sampling characteristics and multi parameters relationships of the radar data are analyzed and presented. Based on the grey correlation analysis method, the correlations of different parameter series are given in Section 4. Finally, in Section 5, we conclude the paper and provide the directions for future work.

## 2. Introduction of the Millimeter-Wave Radar and the Experimental Scenario

In this research, the non-scanning multi-target millimeter-wave radar having FMCW mechanism is used. The core-configuration of the radar are as follows:

**The radar signal processing chip:** NXP-MPC5775K, a 32-bit heterogeneous multi-core microcontroller with two independent e200z7260 cores and an e200z420 core.

**The radio frequency front-end:** NXP-MR2001, a high performance multi-channel 77 GHz radar transceiver chipset. This contributes to better system-level radar solutions with the MPC5775K chip.

**The Antenna system:** The self-developed planar micro strip array antenna, which contains two groups of transmitting antennas and three groups of receiving antennas.

The detailed technical parameters of the radar device are introduced in Table 1.

**Figure 1:**
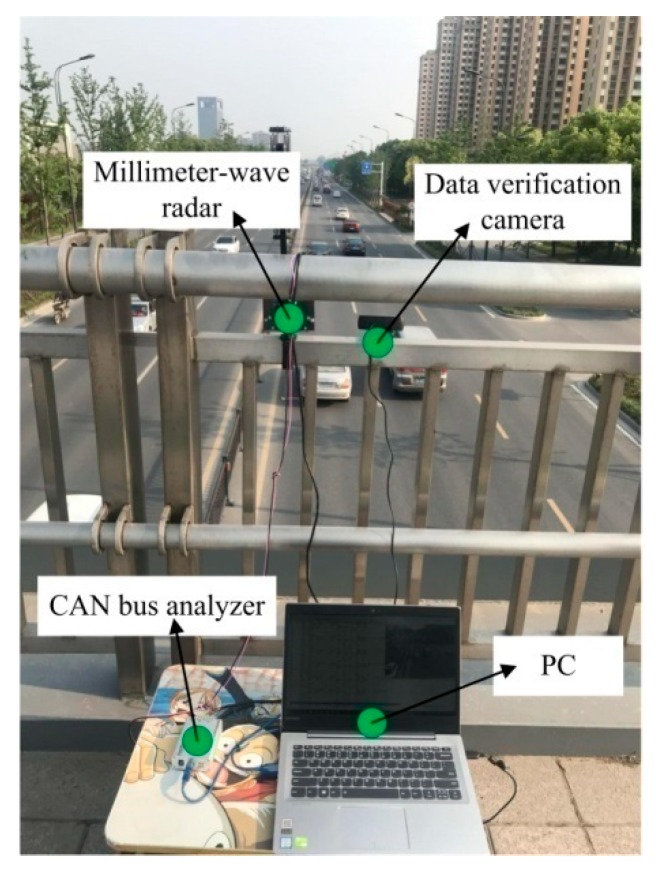
Experimental scenario. CAN: controller area network.

To collect traffic data for further analysis, we have conducted the experiment at Yangzijiang Road, Yangzhou City of China, as the sample case study. We have installed the device on a platform bridge across the experimental road, as is shown in Figure 1. The detailed parameters of the installation and the road section are introduced in Table 2.

As is shown in Figure 1, the millimeter-wave radar traffic flow detection system includes the radar device, controller area network (CAN) bus analyzer, data verification camera and PC for data collection. The radar device detects the information of the target vehicle, including the distance, angle, speed and the RCS energy per 50 ms and output the data using CAN. The CAN bus analyzer interprets the data and transforms it to USB data packet to the PC. In our experiment, the camera is used to verify the data for further off-line analysis.

## 3. Single Parameter Feature Analysis

### 3.1. Sampling Analysis of Distance Values

The millimeter-wave radar used in this research is a non-scanning multi target radar with the FMCW mechanism. The detailed technical parameters of the radar device have been introduced in Table 1.

In millimeter-wave radar, the distance of the target is acquired based on the time delay effect. The radar sends millimeter-wave signal, receives the echo signal and the processing system calculates the target distance further according to the frequency difference and the delay time between the two kinds of signals. The distance measurement algorithm can be expressed by Equation (1).

(1) $$L=\frac{c}{2}\cdot \frac{1}{f}\cdot \frac{f_{b}}{\Delta f}$$

where L is the target distance (m). f denotes millimeter-wave modulation frequency. In this paper, the radar uses the continuous triangular wave. $f_{b}$ is the difference frequency between the original signal and the echo signal. Generally, the longer distance between the radar and the target is, the bigger value of the difference frequency generates. Δf is the bandwidth.

**Figure 2:**
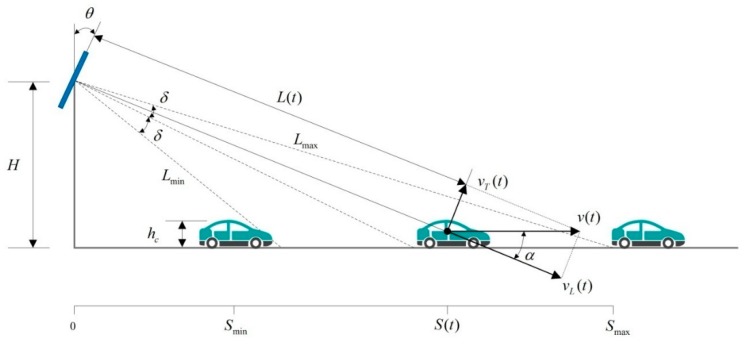
Distance measurement of vehicles.

When the radar is installed above the road, the distance of the vehicles can be detected as shown in Figure 2.

In Figure 2, H is the height of the radar device (m). θ is the pitch angle of the radar device. δ is the pitch angle of the radar signal. $h_{c}$ is height of the vehicle (m). v(t) is the horizontal moving velocity of the vehicle (m/s). $v_{L}(t)$ is the radial velocity and the direction of which is the same as the radar wave transmitting direction. α is the included angle between the radial velocity and the horizontal directions.

Based on Figure 2, the theoretical shortest distance that the radar can measure is:

(2) $$L_{\min\;}=\frac{H-h_{c}}{\cos(90-\theta -\delta )}$$

And the theoretical farthest distance is:

(3) $$L_{\max\;}=\frac{H}{\cos(90-\theta +\delta )}$$

In Figure 2, in essence, $v_{L}(t)$ denotes the component of the vehicle velocity at the radar wave transmitting direction. The value of $v_{L}(t)$ can be calculated by Equations (4) and (5).

(4) $$v_{L}(t)=v(t)\cos\alpha$$

(5) $$v_{L}(t)=v(t)\frac{\sqrt{L^{2}(t)-H^{2}\;}}{L(t)}$$

The hypothesis for further macroscopic data sampling characteristics analysis is described as follows. Considering that a vehicle moves at a constant velocity *V* m/s, where the actual moving speed for a single vehicle is not represented as hypothetical velocity but rather general traffic flow rate in a short term which is very close to the actual scenario.

Considering the measuring distance as independent variable, the derivative of Equation (5) can be shown in Equation (6).

(6) $${v^{\prime }}_{L}(L(t))=\frac{VH^{2}\;}{L^{2}(t)\sqrt{L^{2}(t)-H^{2}}}$$

**Figure 3:**
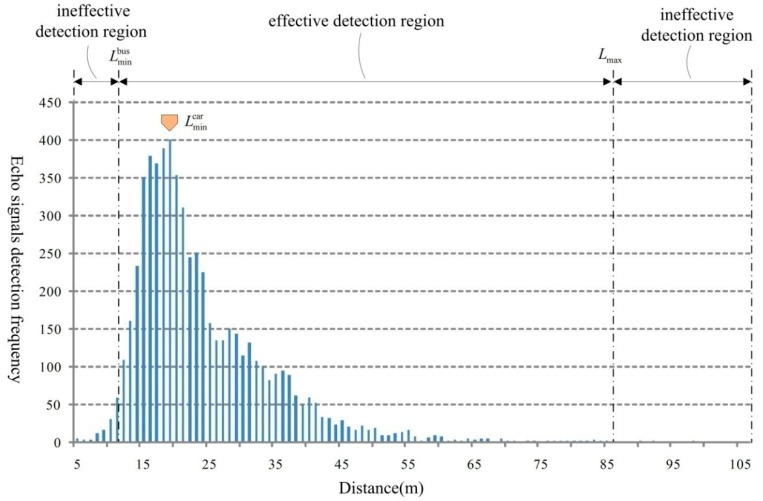
Distribution of the number of times for distance measurement.

In Equation (6), ${v^{\prime }}_{L}(L(t))>0$ and the function established with the component of the vehicle velocity at the radar wave transmitting direction as dependent variable and the measuring distance as the independent variable monotonically increases. In other words, the radial velocity increases when the vehicle moves further away from the radar. Theoretically, under the same sampling frequency, there is an inverse correlation between the radial velocity of the target and the number of times for detecting echo signal. In the experiment, the number of times for detecting echo signal under different distance values is provided in Figure 3.

In our experiment, the pitch angle of the radar device $\theta =12^{{}^{\circ}}$ and the pitch angle of the radar signal $\delta =7^{{}^{\circ}}$. According to Equations (2) and (3), we calculate the minimum measuring distance for big vehicle (the average height values 3.5 m, such as the bus), $L_{\min}^{\mathrm{bus}}=12.3$ m and the minimum measuring distance for small vehicle (the average height values 1.5 m, such as the car), $L_{\min}^{\mathrm{car}}=18.4$ m and the maximum measuring distance that $L_{\max}=86$ m. For the $L_{\max}$, there is no need to distinguish buses or cars since the distance measuring values are without significant differences. As is shown in Figure 3, there are two kinds of regions, the effective detection region (where the measuring distance values are bigger than $L_{\min}^{\mathrm{bus}}$ and smaller than $L_{\max}$) and the ineffective detection region (where the measuring distance values are smaller than $L_{\min}^{\mathrm{bus}}$ or bigger than $L_{\max}$).

In the effective detecting region, when $L>L_{\min}^{\mathrm{car}}$, with the increase of the measuring distance values, the radial velocity of the target vehicle grows and the dwelling time for the vehicle to stay at the certain unit distance reduces. In this case, the sampling gets sparser, which causes the radar data containing less useful information for describing actual vehicle driving status. As is shown in Figure 3, there is a dramatic downward tendency for the number of times for detecting vehicles. However, it is worth to mention that, the frequency at $L_{\min}^{\mathrm{bus}}$ is much less than the top. The reason is that the share of buses in the actual scenario is very low.

In ineffective detection region, the detecting vehicles are beyond the theoretical effective range of the radar. This problem is mainly caused by the side lobe interference. The data in these regions should be rejected for further vehicle analysis.

### 3.2. Sampling Analysis of Angle Values

**Figure 4:**
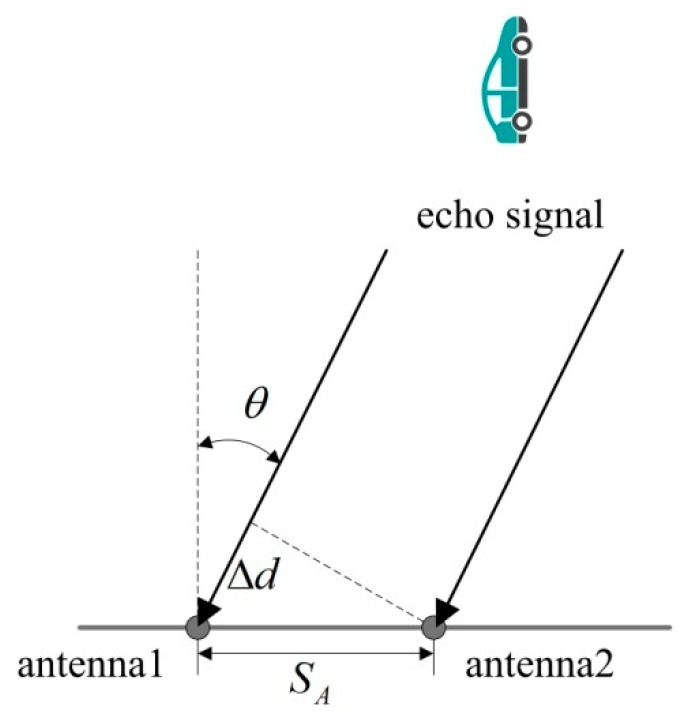
Principle of the radar angle measurement.

In this work, the radar calculates the target angle using the phase difference method based on the linearity of the radio wave propagation and the directivity of the antenna beam. Different antennas receive the same echo signal and the processing system analyzes the phase differences for vehicle angle calculation, as is shown in Figure 4.

The target angle is calculated by Equation (7).

(7) $$\theta =\arcsin(\frac{\lambda \cdot \Delta \phi \;}{2\pi \cdot S_{A}})$$

where θ is the angle of the target vehicle. λ is the wavelength of the radar wave. $S_{A}$ is the antenna distance. Δϕ is the phase difference for antennas receiving the echo signal, which is detected using a phase-comparison circuit.

**Figure 5:**
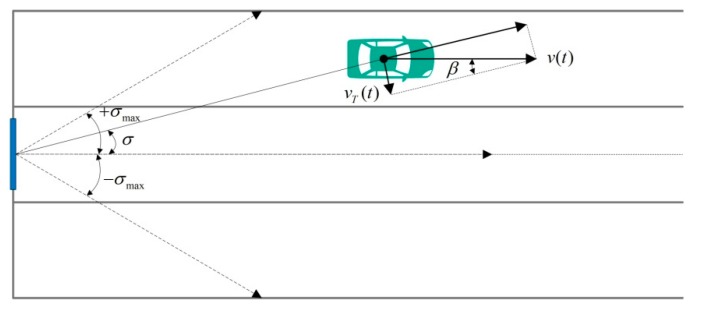
Angle measurement of vehicles.

In the traffic flow detection scenario, the angle measurement can be shown in Figure 5.

**Figure 6:**
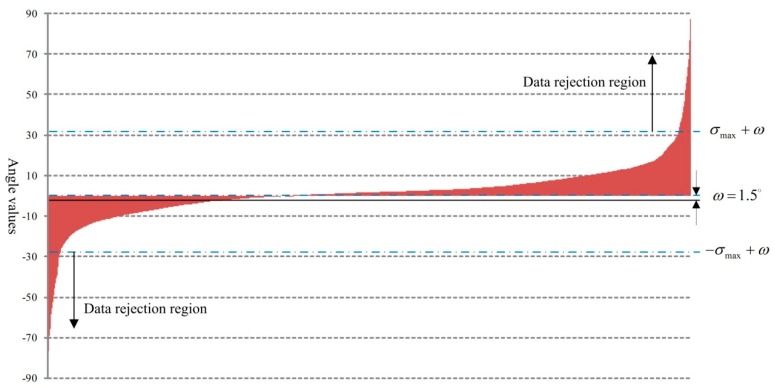
Angle distribution.

In Figure 5, σ is the angle of the target vehicle. $\sigma_{\max}$ is the maximum measurement angle and $-\sigma_{\max}$ is the minimum. We collect the angle data for all vehicles in the given experimental setup and statistical results obtained are shown in Figure 6.

In our experiment, the max angle measurement range is $\pm 30^{{}^{\circ}}$. There is an angle error ω between the vertical direction of the radar surface and the straight direction of traffic and $\omega =1.5^{{}^{\circ}}$, as is shown in Figure 6. From the figure, we can also find that there is a mass of data out of range [$30^{{}^{\circ}}+\omega$, $-30^{{}^{\circ}}+\omega$]. This false detection is calculation error or caused by the side lobe. In further vehicle analysis, the data in these regions should be rejected.

In Figure 5, the $v_{T}(t)$ denotes the tangential component of the vehicle velocity at the radar wave transmitting vertical direction and it is calculated as follows:

(8) $$v_{T}(t)=v(t)\sin\beta =v(t)\sin\sigma =v(t)\frac{l_{lane\;}}{L(t)},\;\sigma >0$$

Similarly, suppose that the vehicle moves at a constant velocity V, considering the measuring distance as independent variable, we obtain the derivative of Equation (8):

(9) $${v^{\prime }}_{L}(L(t))=V(\frac{l_{lane\;}}{L(t)}{)}^{\prime }=-\frac{Vl_{lane}}{L^{2}(t)}$$

When σ>0, ${v^{\prime }}_{L}(L(t))<0$. It implies that the function established with the component of the vehicle velocity at the radar wave transmitting vertical direction as dependent variable and the measuring distance as the independent variable monotonically decreases. The detecting angle value decreases when the vehicle goes further away from the radar. Meanwhile, the tangential velocity component of the vehicle velocity around the radar position also decreases and the dwelling time for the vehicle to stay at certain unit angle increases. In this case, the sampling gets denser. Similarly, when σ<0, the sampling characteristics are symmetric with the case σ>0 based on the symmetry axis of central vertical of the radar surface. As a conclusion, the sampling for angle measurement follows normal distribution in the range of $\left[-\sigma_{\max},\sigma_{\max}\right]$.

**Figure 7:**
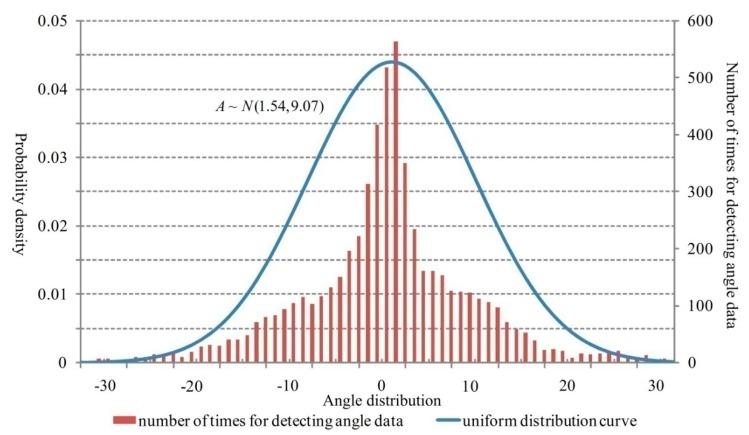
Distribution of the frequency for angle measurement.

The probability distribution for angle data sampling after rejecting the abnormal data is shown in Figure 7. The angle values mostly follow the uniform distribution that effectively verifies the inference mentioned above.

### 3.3. Sampling Analysis of Radar Cross-Section Energy Values

**Figure 8:**
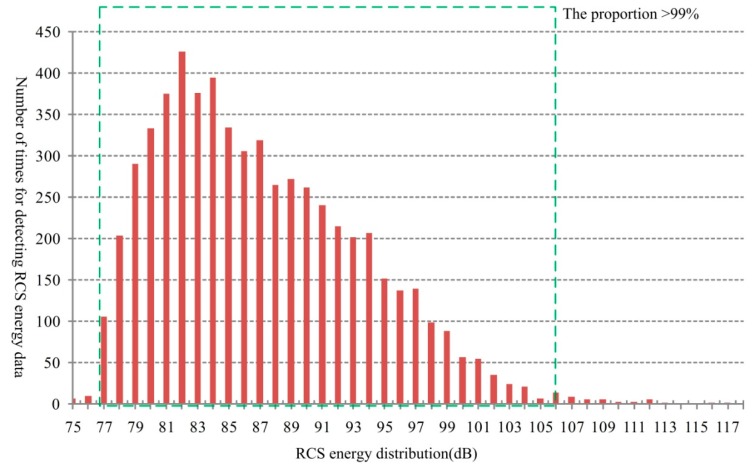
Distribution of the number of times for RCS energy measurement.

**Figure 9:**
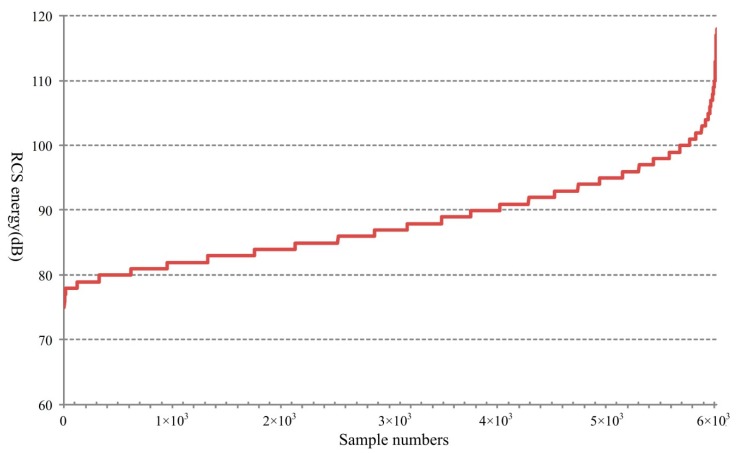
RCS energy order.

In this paper, the RCS energy is calculated by the signal level of the actual echo signal wave which depends on the RCS characteristics of the target itself. When obtaining the analog-to-digital converter (ADC) sampling points of the echo signal, a data conversion is taken for the signal level to RCS energy value, which represents the signal strength reflected by the target. In the experimental road, there are many types of motor vehicles, such as buses, cars, trucks, agitating lorries and others. Figure 8 shows the RCS energy distribution and Figure 9 shows the RCS energy order from the smallest to the largest. For different vehicles, the RCS energy changes between a minimum value 75 dB and a maximum value 118 dB. Besides, the proportion for the RCS energy locates within the range [77, 106] is more than 99%. In Figure 9, we can see that, there is an abrupt increase at the end of the curve for a small sample with high energy values. It is mainly caused by the big vehicles, such as buses, returning high energy in the echo signal.

**Figure 10:**
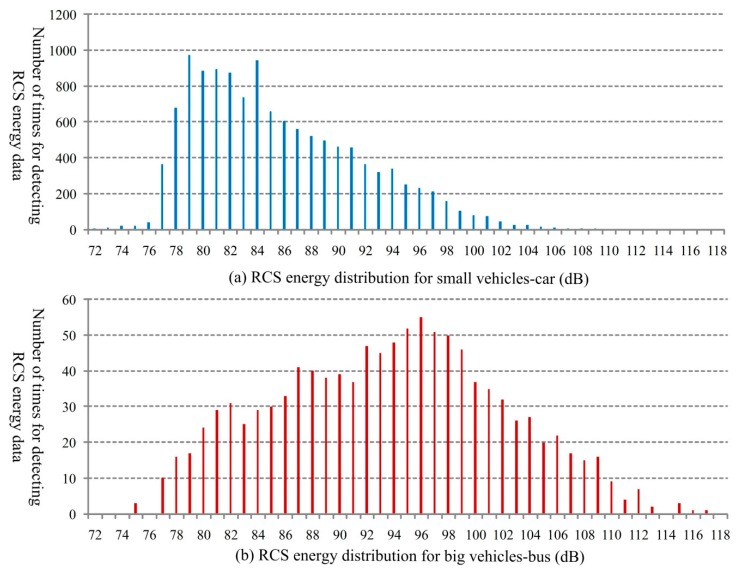
Distribution of the number of times for RCS energy measurement for different types of vehicles.

In order to further analyze the RCS energy characteristics for different type of vehicles, the sampling distribution for the big vehicles (mainly the buses) and the small vehicles (mainly the cars) are shown in Figure 10a,b. From Figure 10, it is evident that, the two types of vehicles cause obvious different RCS energy distributions.

**Figure 11:**
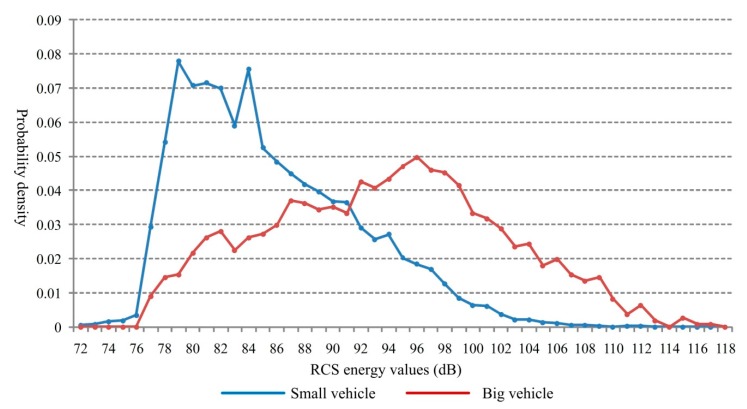
Probability distributions for the two types of vehicles.

Based on Figure 10, the probability distributions for the two types of vehicles are shown in Figure 11.

From Figure 11, the RCS energy presents obvious difference for the two types of vehicles. For the small vehicle, the RCS energy values are concentrated in the low-energy region with an average of 85.6 dB and the big vehicle in high-energy region, with an average of 93.7 dB. According to the probability distribution shown above, the RCS energy can be used to help to identify the target is a big vehicle or a small one.

**Figure 12:**
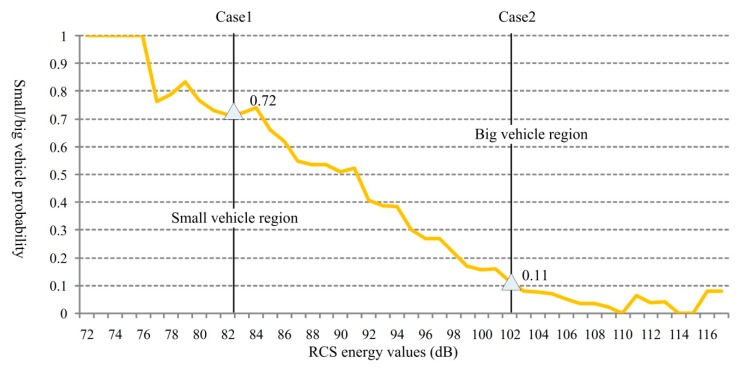
Vehicle type identification based on RCS energy values.

The curve shown in Figure 12 is calculated based on the probability distribution given in Figure 11. In the figure, two regions are formed at the sides of the curve, which can be used for assisting vehicle type identification. For example, in Case1, when the radar receives an echo signal including an 82 dB RCS energy value, the target vehicle may be a small vehicle with a probability of 72% and a big vehicle with a probability of 100% − 72% = 28%. Similarly, in Case2, the target may be a small vehicle with a probability of 11% and a big vehicle with a probability of 100% − 11% = 89% when the RCS energy is 102 dB.

### 3.4. Multiple Parameters Analysis

In actual experiment, there is certain relationship between two different measuring parameters. In this section, the data characteristics between the RCS energy and distance, angle error and distance and angle error and RCS energy are discussed.

#### 3.4.1. Radar Cross Section Energy Distribution under Different Distances

**Figure 13:**
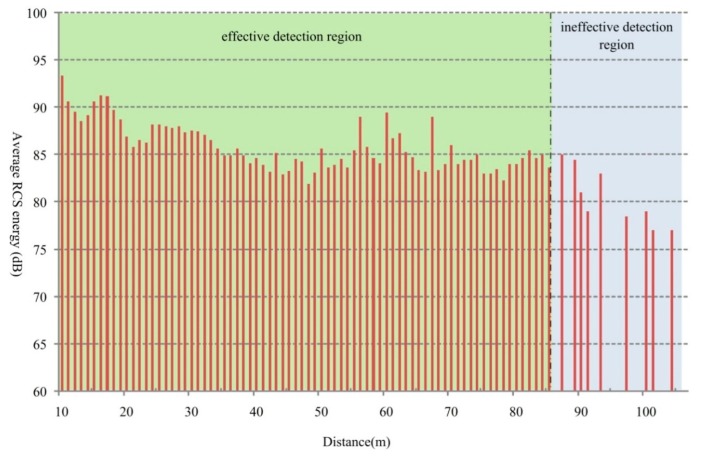
RCS energy distribution under different distances.

The statistical results of the RCS energy under different distances are presented in Figure 13, where the *y*-axis implies the average RCS energy value for small vehicles detected at a certain distance.

As shown in Figure 13, it is easy to find that the average RCS energy presents an overall decreasing trend against the increasing measuring distance in the effective detecting region. This is because the farther the vehicle moves away from the radar, the more dispersive the radar wave transmits and the lower energy that the target vehicle reflects. Especially, when the target distance is farther than 86 m, there is high missing detection. This also matches well with the analysis results described in Section 3.1. 

#### 3.4.2. Angle Error Distribution under Different Distances

**Figure 14:**
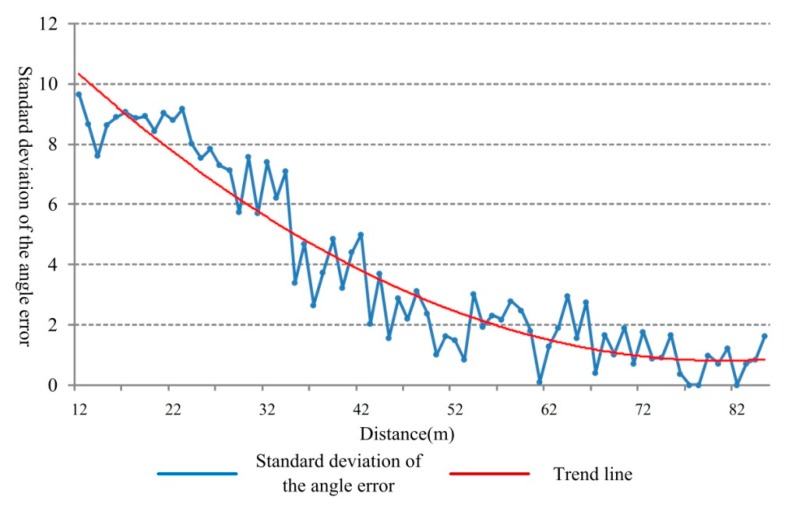
Standard deviation distribution of angle error under different distances.

Taking the middle lane as an example, the detecting angle error under different distances and the results are shown in Figure 14. The *y*-axis implies the standard deviation of the angle error values which is calculated based on the assumption that the vehicle moves along the center line of the lane and the theoretical angle is 0.

From Figure 14, it is evident that, with the increase of the distance, the measuring angle tends to be stable and the angle error standard deviation gets smaller. However, there is still relatively huge error in the target detection process. There are two reasons for this trend. Firstly, the radar used is a non-scanning millimeter-wave radar where the angle measurement accuracy is relatively low. Secondly, when the target vehicle is near to the radar and the trajectory does not strictly follow the center line of the lane and it will cause observable error which is reflected in beginning of the curve shown in Figure 14.

#### 3.4.3. Angle Error Distribution under Different Radar Cross Section Energy Values

**Figure 15:**
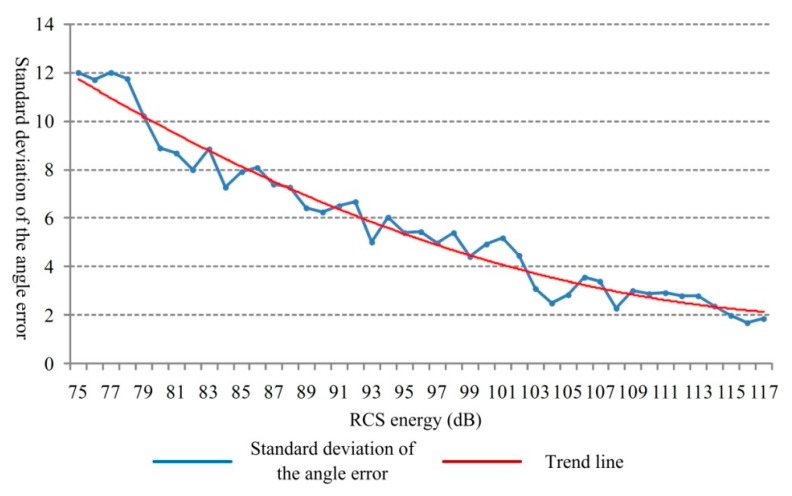
Angle error standard deviation distribution under different RCS energy values.

Taking the middle lane as an example, the detecting angle error under different RCS energy values are shown in Figure 15.

As shown in Figure 15, there is negative correlation relationship between the angle error standard deviation distribution and the RCS energy. With the increase of the RCS energy, the angle error standard deviation gets smaller. When the RCS energy is higher than 100 dB, the angle error standard deviation is below 4°. Hence, the high RCS energy provides suitable criteria for the target angle discrimination.

## 4. The Correlation Analysis for Parameter Series

In Section 3, the sampling characteristics of the single parameter and the relationship between two different parameters are discussed. Based on the above analysis, this section further studies the inherent correlation for the multiple dimension parameter series using a grey correlation analysis (GRA) method.

### 4.1. Correlation Analysis for Multiple Dimension Radar Parameter Series Based on GRA

The grey correlation analysis is an important research area in grey system theory. It uses the linear interpolation method to translate the discrete observed value of behavior series to a piecewise continuous broken line and further estimates the correlation of different series according to the geometric curve shapes of the corresponding broken lines.

Suppose the series of the characteristic behaviors are:

(10) $$X_{0}(k)=(x_{0}(1),x_{0}(2),\cdots ,x_{0}(n))$$

The series of relevant factors are:

(11) $$X_{i}(k)=(x_{i}(1),x_{i}(2),\cdots ,x_{i}(n))$$

The displacement difference of series $X_{0}$ and $X_{i}$ at the point k can be expressed by Equation (12).

(12) $$\Delta x_{0i\;}(k)=x_{0}(k)-x_{i}(k)$$

Further, $\left|\Delta x_{0i}(k)\right|$ is the absolute difference which represents the close degree for the two series at the point k. The smaller the value of $\left|\Delta x_{0i}(k)\right|$ is, the closer the two lines are.

The correlation degree for $X_{i}$ and $X_{0}$ is expressed by Equation (13).

(13) $$\gamma (X_{0},X_{i})=\sum_{k=1\;}^{n}\omega_{k}\gamma (x_{0}(k),x_{i}(k))$$

In Equation (13), $\omega_{k}$ is the weight. $\gamma (x_{0}(k),x_{i}(k))$ is thecorrelation coefficient, which is calculated by Equation (14).

(14) $$\gamma (x_{0}(k),x_{i}(k))=\frac{\underset{i}{\min\;}\underset{k}{\min}|\Delta x_{0i}(k)|+\rho \;\underset{i}{\max}\;\underset{k}{\max}|\Delta x_{0i}(k)|}{\left|\Delta x_{0i}(k)|+\rho \;\underset{i}{\max}\;\underset{k}{\max}|\Delta x_{0i}(k)\right|}$$

where $\underset{i}{\min}\;\underset{k}{\min}|\Delta x_{0i}(k)|$ is the minimum difference and $\underset{i}{\max}\;\underset{k}{\max}|\Delta x_{0i}(k)|$ is the maximum difference. ρ is the resolution ration and ρ∈[0,1].

Let $\bar{\Delta x_{0i}}$ denote the average of the all absolute value difference, as expressed by Equation (15).

(15) $$\bar{\Delta x_{0i\;}}=\frac{1}{n\cdot m}\sum_{i=1}^{m}\sum_{k=1}^{n}\left|\Delta x_{0i}(k)\right|$$

And,

(16) $$\in_{\Delta }=\frac{\bar{\Delta x_{0i\;}}}{\underset{i}{\max}\;\underset{k}{\max}|\Delta x_{0i}(k)|}$$

The resolution ration ρ values:

(17) $$\left\{ \begin{array}{l} \in_{\Delta }\leq \rho \leq 1.5\in_{\Delta },\;when\;\in_{\Delta }\geq \frac{1}{3}\;\\ 1.5\in_{\Delta }\leq \rho \leq 2\in_{\Delta },\;when\;\in_{\Delta }<\frac{1}{3} \end{array}\right.$$

As elaborated in [36], the above-mentioned method can decrease the influence of abnormal values on the grey correlation degree calculation.

In our experiment of millimeter-wave radar for traffic flow detection, the vehicle travels in one direction only, hence the target distance parameter is absolutely monotonic. As introduced in Section 2, the distance measuring error of the radar device is ±1 m, with high accuracy. Besides, both the angle and RCS energy characteristics are closely related with the distance. Hence, we choose the target measuring distance as the characteristic behavior series, the angle and RCS energy as the relevant factors, to build the GRA model.

According to the analysis described in Section 3.1, with the increase of the measuring distance values, the radar data contains less useful information for describing actual vehicle driving status. In order to make the grey correlation analysis results more reasonable, the data which is near the radar position should be assigned a higher weight. Based on the sampling characteristic given in Figure 2, we use the normalized Gaussian function to assign values to the weight coefficient.

(18) $$\omega (i)=\frac{\omega^{\prime }(i)\;}{\sum_{i=1}^{n}\omega^{\prime }(i)}$$

where,

(19) $$\omega^{\prime }(i)=e^{-\tau |L(i)-L_{\min\;}{|}^{2}},\;L_{\min}\leq L(i)\leq L_{\max}$$

In Equation (19), τ is the accommodation coefficient. Suppose that the minimum weight confidence level is σ when $L=L_{\max}$, the accommodation coefficient is calculated as follows:

(20) $$e^{-\tau |L_{\max\;}-L_{\min}{|}^{2}}\leq \sigma$$

(21) $$\tau \geq -\frac{\ln\sigma \;}{{\left(L_{\max}-L_{\min}\right)}^{2}}$$

**Figure 16:**
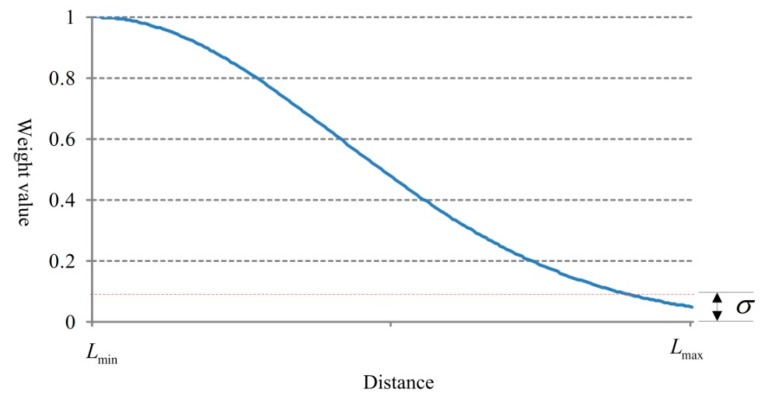
Weight distribution of the parameter series.

The weight distribution is shown in Figure 16.

Finally, the average of correlation coefficients of all data in the parameter series is taken as the index for describing the grey-relation degree, as shown in Equation (22).

(22) $$r_{i}=\frac{1}{m}\sum_{k=1\;}^{m}\gamma (X_{0},X_{i})\;i=1,2$$

In Equation (22), i=1, representing the square deviation of the angle error. i=2, representing the RCS energy.

### 4.2. Data Analysis

**Figure 17:**
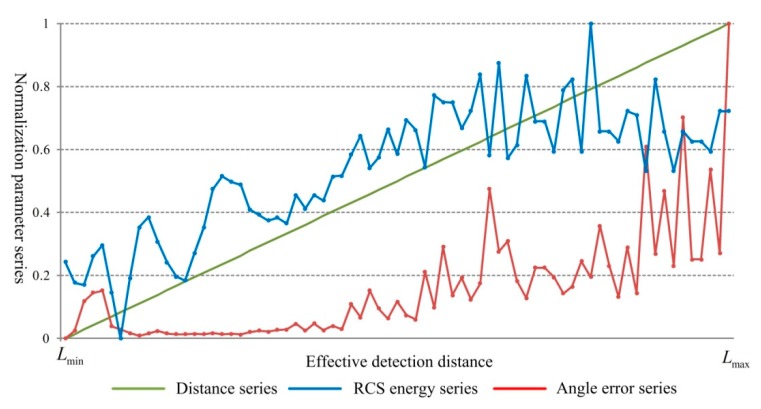
Data series variation.

As described above, we choose the target measuring distance as the characteristic behavior series, the angle and RCS energy as the relevant factors to build the GRA model. From the analysis results shown in Section 3.4, both the angle error and the RCS energy show negative correlation with the increase of measuring distance. In order to make different kinds of data changes consistent, the reciprocals of the angle error and the RCS energy are firstly calculated for preprocessing. Based on this, normalization operation is taken to make the data change in the range of [0, 1]. The data series are shown in Figure 17.

**Figure 18:**
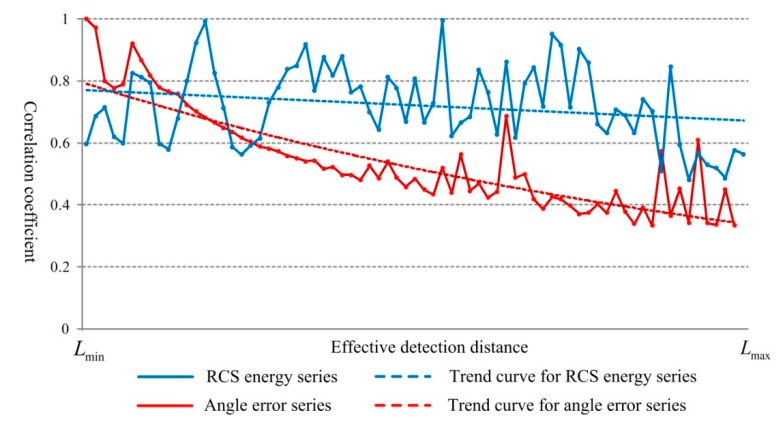
Variations of the correlation coefficients.

The variations of the correlation coefficients are shown in Figure 18.

From the results obtained, as the target vehicles move further away from the radar, the measuring distance increases and the correlation coefficients of both the angle error and the RCS energy show an overall downward trend. This indicates that the radar data detected at close range is highly reliable. In addition, by Equation (22), the grey relational degree of RCS energy is 0.72 and the value of angle error is 0.54. Compared with the angle measurement, the RCS energy distribution presents a much steady trend as the distance increases. The angle accuracy is affected by the antenna performance. For the non-scanning radar which we used in this study, the angle measurement exists relatively high and having random error, presenting a weaker correlation behavior.

## 5. Conclusions

In this paper, the data features are analyzed for the non-scanning multi target millimeter-wave radar used in traffic flow detection, with single parameter sampling, multiple parameters and parameter series correlation analysis. The main conclusions are summarized as follows:

- Based on the application scenario, the effective detecting region of the millimeter-wave radar is calculated and further the data sampling characteristics are analyzed in the region. At a certain close range, the sampling is dense and contains more useful information for further research.
- Subject to the antenna performance, there are obvious errors for angle measurement and the errors show certain random characteristics. Hence, the angle information cannot be used alone for further applications, such as lane recognition. In addition, when the RCS energy is high, the angle error is relatively low, which provides a method to combine the RCS energy and the angle information together for corresponding applications.
- The RCS energy presents a downward trend as the vehicle moves away from the radar. For different types of vehicles, the RCS energy distributions have certain differences, which can be used for identifying small or big vehicle targets. In this paper, a simple method for target type identification is also proposed.
- Combining with the second item mentioned above, from the point of view of the overall sampling series, the RCS energy presents a much higher correlation degree compared to the angle as the distance increases.

These conclusions can provide effective reference for the further applications where millimeter-wave radar is used in the traffic flow detection. Based on the analysis, the future work mainly focuses on the following aspects:

- The data feature analysis for stationary vehicles using the stationary target detection function of the FMCW millimeter-wave radar.
- Further traffic status identification research based on the data features acquired, for example, multi-target trajectories tracking, traffic volume calculation and parking event judgment.

## Figures and Tables

**Table 1:** Technical parameters of the millimeter-wave radar.

Parameter	Value
Antenna type	non-scanning
Frequency range	76–77 GHz
Distance measurement range	0–120 m
Distance accuracy	±1 m
Velocity measurement range	−70 m/s–+70 m/s
Velocity accuracy	±0.5 m/s
Angle detection range	±30°
Pitch angle	±7°
Max measurement target numbers	32
Detection cycle	50 ms

**Table 2:** Parameters of the installation and the road section.

Parameter	Value
Height of the radar	7.5 m
Pitch angle of the radar surface	12°
Number of lanes	3
Lane width	3.5 m
